# Novel Prefrontal Synthesis Intervention Improves Language in Children with Autism

**DOI:** 10.3390/healthcare8040566

**Published:** 2020-12-16

**Authors:** Andrey Vyshedskiy, Edward Khokhlovich, Rita Dunn, Alexander Faisman, Jonah Elgart, Lisa Lokshina, Yuriy Gankin, Simone Ostrovsky, Lauren deTorres, Stephen M. Edelson, Petr O. Ilyinskii

**Affiliations:** 1 Biology Department, Boston University, Boston, MA 02215, USA; 2 ImagiRation, Boston, MA 02135, USA; ritachka@gmail.com (R.D.); jonah.elgart@gmail.com (J.E.); lokshin.lisa@gmail.com (L.L.); Simone.Ostrovsky@gmail.com (S.O.); ldetorres@colgate.edu (L.d.); 3 Independent Researcher, Newton, MA 02459, USA; edpiter@gmail.com; 4 Independent Researcher, New York, NY 10002, USA; afaisman@gmail.com; 5 Quantori, Cambridge, MA 02139, USA; yuriy.gankin@quantori.com; 6 Autism Research Institute, San Diego, CA 91911, USA; director@autism.com; 7 Independent Researcher, Cambridge, MA 02140, USA; p.ilyinskii@verizon.net

**Keywords:** autism, ASD, language delay, language therapy, imagination, theory of mind

## Abstract

Prefrontal synthesis (PFS) is defined as the ability to juxtapose mental visuospatial objects at will. Paralysis of PFS may be responsible for the lack of comprehension of spatial prepositions, semantically-reversible sentences, and recursive sentences observed in 30 to 40% of individuals with autism spectrum disorder (ASD). In this report we present data from a three-year-long clinical trial of 6454 ASD children age 2 to 12 years, which were administered a PFS-targeting intervention. Tablet-based verbal and nonverbal exercises emphasizing mental-juxtaposition-of-objects were organized into an application called *Mental Imagery Therapy for Autism* (MITA). The test group included participants who completed more than one thousand exercises and made no more than one error per exercise. The control group was selected from the rest of participants by a matching procedure. Each test group participant was matched to the control group participant by age, gender, expressive language, receptive language, sociability, cognitive awareness, and health score at first evaluation using propensity score analysis. The test group showed a 2.2-fold improvement in receptive language score vs. control group (*p* < 0.0001) and a 1.4-fold improvement in expressive language (*p* = 0.0144). No statistically significant change was detected in other subscales not targeted by the exercises. These findings show that language acquisition improves after training PFS and that a further investigation of the PFS-targeting intervention in a randomized controlled study is warranted.

## 1. Introduction

Full command of complex language depends on understanding of vocabulary as well as on the mechanism of juxtaposition of mental visuospatial objects into novel combinations, called prefrontal synthesis (PFS) [1]. Without developed PFS, it is impossible to understand the difference between sentences with identical words and grammar, such as “the cat on the mat” and “the mat on the cat.” Most people assume innate PFS abilities in all individuals. Scientific evidence, however, suggests a more intricate story. While propensity toward PFS is innate in humans, acquisition of PFS seems to be the function of using recursive language in early childhood [2,3,4,5]. The myelination of frontoposterior fiber tracts mediating PFS [6] depends on early childhood conversations [7,8]. In the absence of normal recursive conversations, children do not fine-tune these neurological connections and, as a result, do not acquire PFS [9]. The autism community refers to the phenomenon whereby individuals cannot combine disparate objects into a novel mental image as *stimulus overselectivity*, or *tunnel vision*, or *the lack of multi-cue responsivity* [10,11,12,13]. Failure to juxtapose mental objects, called *PFS paralysis*, results in a lifelong inability to understand spatial prepositions, semantically-reversible sentences (e.g., “the mat on the cat”), and recursion. Among individuals diagnosed with autism spectrum disorder (ASD), the prevalence of PFS paralysis is 30 to 40% [14] and may be as high as 60% among children enrolled into special ASD schools [15].

We hypothesized that early PFS-targeting intervention can improve language ability in children with ASD. Accordingly, we designed various developmental activities, all of which follow a systematic approach to train PFS verbally as well as outside of the verbal domain [16,17,18,19]. To make these activities dynamic and attractive to children, we organized them into an application called *Mental Imagery Therapy for Autism* (MITA). The MITA app was released in 2015 and quickly rose to the top of the “autism apps” charts [16].

MITA verbal activities start with simple vocabulary-building exercises and progress toward exercises aimed at higher forms of language, such as noun–adjective combinations, spatial prepositions, recursion, and syntax [17]. For example, a child can be instructed to *select the {small|large} {red|blue|green|orange} ball*, or to *put the cup {on|under|behind|in front of} the table*. All exercises are deliberately limited to as few nouns as possible since the aim is not to expand a child’s one-word vocabulary, but rather to teach him/her to integrate mental objects in novel ways by utilizing PFS [17].

MITA activities outside of the verbal domain aim to provide the same PFS training visually through implicit instructions as has been described in Dunn et al. [18]. For example, a child can be presented with two separate images of a train and a window pattern, and a choice of complete trains. The task is to find the correct complete train and to place it into the empty square. This exercise requires not only attending to a variety of different features in both the train and its windows, but also combining two separate pieces into a single image (in other words, mentally *integrating* separate train parts into a single unified gestalt). As levels progress, the exercises increase in difficulty, requiring attention to more and more features and details. Upon attaining the most difficult levels, the child must attend to as many as eight features simultaneously. Previous results from our studies have demonstrated that children who cannot follow the explicit verbal instruction can often follow an equivalent command implicit in the visual set-up of the puzzle [17].

The idea of using nonverbal PFS exercises in children with developmental delay dates back to Piaget, Vygotsky, and Luria [20]. In his famous twin study, Luria used educational games developed from a set of blocks to try to improve one twin’s PFS ability. Using blocks, he designed two types of learning activities: (1) build-from-elements, and (2) build-from-model. The ‘build-from-elements’ activity involved building a structure per design that indicated the contours of individual blocks necessary for construction. The ‘build-from-model’ activity indicated the overall outline of the final structure but did not specify what blocks to use (i.e., the design did not have contours of individual blocks indicated on it). Luria then studied ten children (5 pairs of identical twins; age 5 years). One twin of each pair followed a 2.5-month program using strategy #1 and the other using strategy #2. When tested at the end of the program, both twins in a pair were equally good at discriminating elementary figures and concentrating, but the twin following program #2 was superior in both PFS and language; he planned more, and had a better sense of the relation of a block to the whole structure. Program #2 twins were also *more articulate* when identifying differences between their structure and the model they were working towards [20].

The use of tablet computers significantly enhances the range and adaptability of nonverbal exercises. MITA nonverbal exercises are limitless in variations, therefore avoiding routinization. Each activity is dynamic, quickly adjusting to the child’s exact ability level. All activities are disguised as games that engage children. Furthermore, many MITA verbal modules start with nonverbal levels. For example, the *Prepositions on/under* and the *Prepositions in front/behind* games have each 30 introductory nonverbal levels (whereby objects arrangement is guided by picture-example alone), 30 intermediate levels (where objects arrangement is guided by both picture-example and verbal instruction), and 30 advanced levels, where objects arrangement is guided by verbal instruction alone. Since nonverbal exercises are easier to children with ASD [17], MITA starts with nonverbal exercises and adds up verbal exercises slowly in order to keep the fine balance between being engaging and challenging.

PFS is an internal, subjective function that does not easily manifest itself to evaluators [15]. Unlike vocabulary acquisition, PFS takes years to develop in an ASD child. With a long intervention time and in the absence of accepted PFS tests, a multi-year randomized controlled trial (RCT) of a PFS-targeting intervention is an arduous proposition. We were not able to gain financial support for an RCT and therefore resolved to conduct a simpler self-funded observational trial.

We have previously described a framework for investigating targeted interventions for ASD children epidemiologically, whereby caregivers submit multiple assessments longitudinally [21]. Using the comprehensive 77-question Autism Treatment Evaluation Checklist (ATEC) [22] over the period of several years we have demonstrated significant differences in outcomes along several parameters [21]. Younger children improved more than the older children in all four ATEC subscales—Language, Sociability, Cognitive awareness, and Health. Children with milder ASD demonstrated a higher improvement in the language subscale than children with more severe ASD. No difference between females vs. males was registered in all cohorts studied.

In this report we apply the same framework to study the PFS intervention called MITA in children age 2 to 12 years. The data collected over five years show greater language improvement in MITA-engaged children compared to controls matched by age, gender, expressive language, receptive language, sociability, cognitive awareness, and health at the first evaluation.

## 2. Methods

### 2.1. MITA Exercises

**Figure 1:**
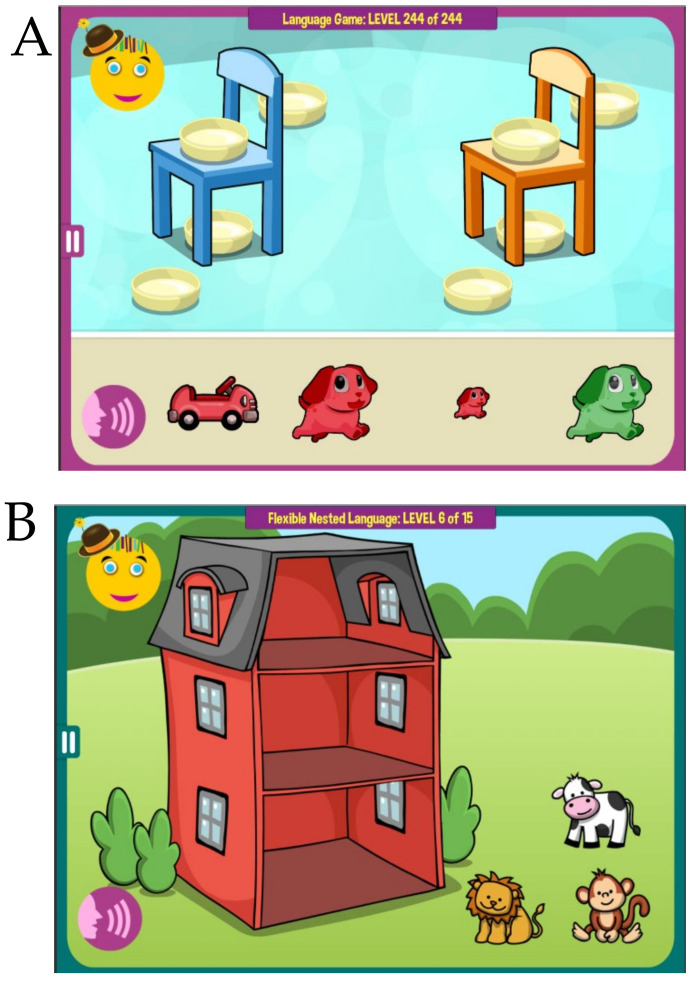
Examples of the *Mental Imagery Therapy for Autism* (MITA) app verbal exercises. (**A**) A child is instructed to put the *large red dog behind the orange chair*. (**B**) A child is instructed: *Imagine*. *The lion lives above the monkey and under the cow. Take animals home*. Note that animals cannot be dragged to their apartments during instructions, encouraging a child to imagine animals’ correct positions in the mind.

MITA includes both verbal and nonverbal exercises aiming to develop voluntary imagination ability [23] in general, and prefrontal synthesis (PFS) ability in particular [15]. The fidelity, validity and reliability of MITA was discussed in detail in Refs. [16,17,18,19]. MITA verbal activities use higher forms of language, such as noun–adjective combinations, spatial prepositions, recursion, and syntax [17] to train PFS (e.g., a child can be instructed to put the *large red dog behind the orange chair*, Figure 1A, or *identify the wet animal* after *the lion was showered by the monkey*, or *take animals home* following an explanation that *the lion lives above the monkey and under the cow*, Figure 1B). In every activity a child listens to a short story and then works within an immersive interface to generate an answer. Correct answers are rewarded with pre-recorded encouragement and flying stars. To avoid routinization, all instruction sentences are generated dynamically, assembled from individual pre-recorded words. For example, in Figure 1B, the instruction rotates through many different animals—*the* {*lion|bunny|lion|dog|cat*} *lives above the* {*monkey|horse|bear|elephant|giraffe*} *and under the* {*cow|duck|dinosaur|turtle|sheep|crocodile*}—and many different sentence structure variations. Collectively, the verbal activities have over 10 million different instructions, and therefore a child will almost never hear the same instruction once again.

**Figure 2:**
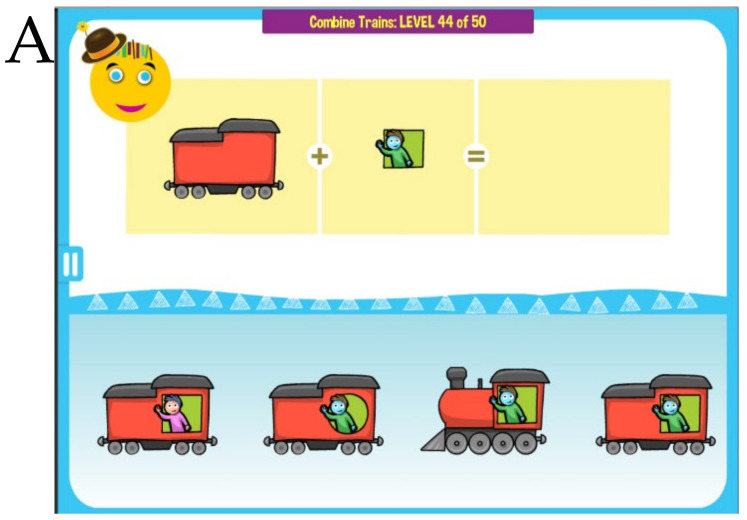

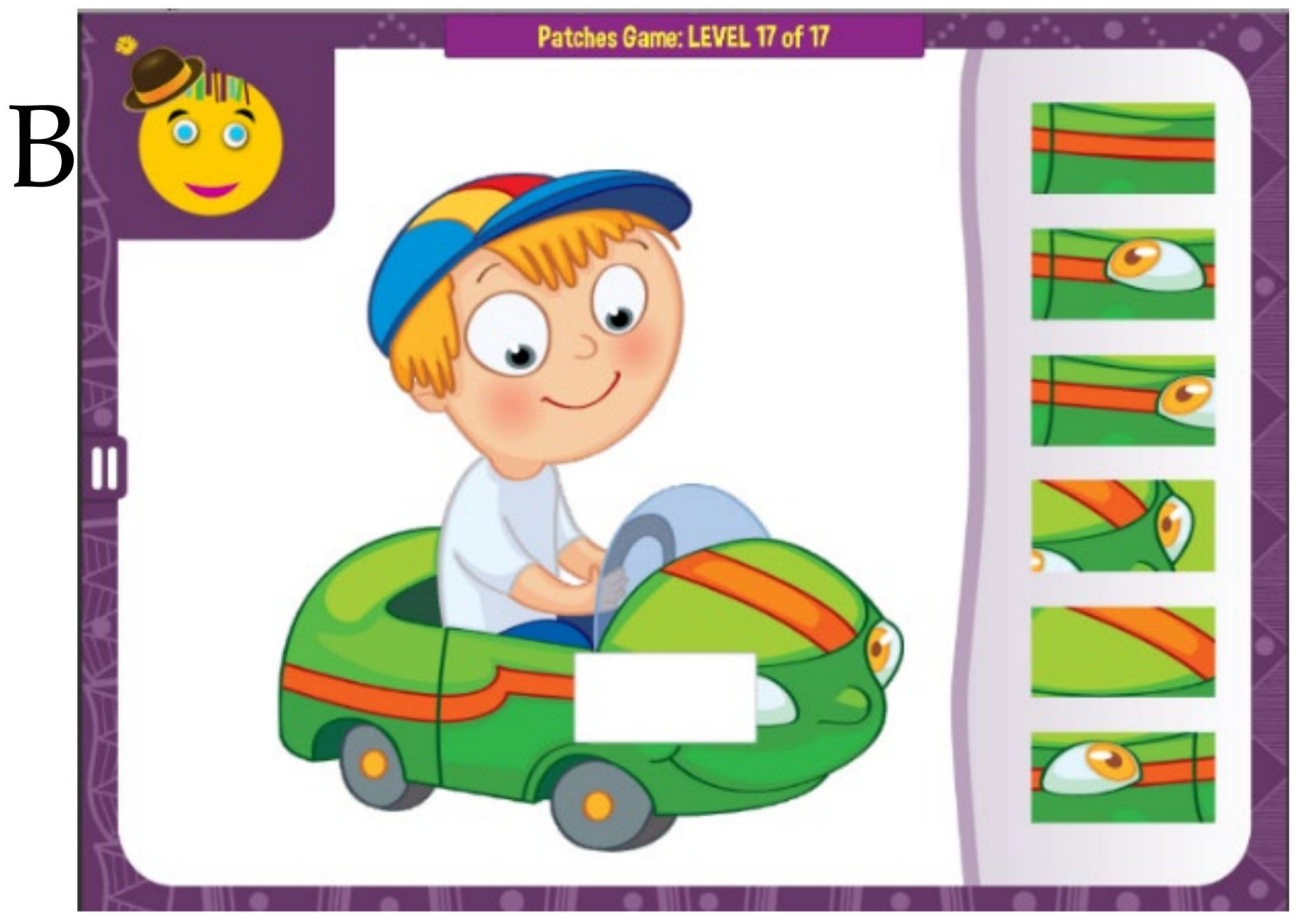
Examples of MITA nonverbal exercises. (**A**) Implicit instruction: *Find the correct train.* (**B**) Implicit instruction: *Find the correct patch.*

MITA nonverbal activities aim to provide the same PFS training visually through implicit instructions [18]. For example, a child can be presented with two separate images of a train and a window pattern, and a choice of complete trains. The task is to find the correct complete train. The child is encouraged to avoid trial-and-error and integrate separate train parts mentally, thus training PFS, Figure 2A. Different games use various tasks and visual patterns to keep the child engaged, Figure 2B. Most puzzles are assembled dynamically from multiple pieces in such a way that they never repeat themselves.

**Figure 3:**
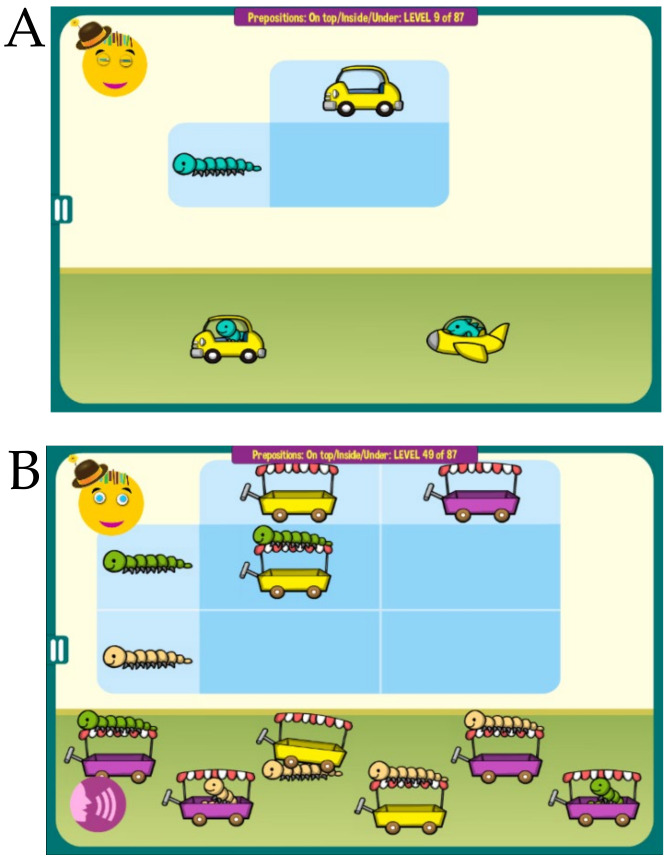
Examples of an MITA game that teaches spatial prepositions *above* and *under*. (**A**) The game starts with the implicit instruction to combine an animal and a vehicle. (**B**) At more difficult levels a child must notice the correct positioning of the animal (*above* or *under*), which is announced verbally and also indicated by a visual clue in the top left corner of the matrix (in this figure the visual cue is the caterpillar on top of the cart). At the most difficult levels (not shown) the visual clue is hidden and a child must rely on the verbal instruction alone.

MITA also includes a number of hybrid activities that start children on easier nonverbal exercises and then gradually increase in difficulty, first to a combination of a verbal instruction and a visual clue and later to a verbal instruction alone, Figure 3. Collectively, MITA activities are designed to last for approximately 10 years.

### 2.2. Participants

The MITA app was made available gratis at all major app stores in September 2015. Once the app was downloaded, the caregiver was asked to register and to provide demographic details, including the child’s diagnosis and age. Caregivers consented to anonymized data analysis and completed the Autism Treatment Evaluation Checklist (ATEC) [22], as well as an evaluation of the receptive language using the Mental Synthesis Evaluation Checklist (MSEC) [24]. The first evaluation was administered approximately one month after the first use of MITA and once 100 puzzles had been completed. The subsequent evaluations were administered at approximately three-month intervals. To enforce regular evaluations, the MITA app became unusable at the end of each three-month interval and parents needed to complete an evaluation to regain its functionality.

From this pool of potential study participants, we selected participants based on the following criteria:

(1) Consistency: Participants must have filled out at least three ATEC evaluations and the interval between the first and the last evaluation must have been six months or longer;

(2) Diagnosis: The subjects must have self-reported their diagnosis as ASD. The ASD diagnosis was not verified directly, as we cannot ask participants to submit documentation. However, ATEC scores support ASD diagnosis. Average initial ATEC total score in the test group was 68.26 ± 24.14, and 67.73 ± 23.37 in the control group (Table 1), which corresponds to moderate to severe ASD as delineated in Ref. [25] and Table 2;

(3) Maximum age: Participants older than twelve years of age were excluded from this study;

(4) Minimum age: Participants who completed their first evaluation before the age of two years were excluded from this study.

After excluding participants that did not meet these criteria, there were 6454 total participants, from whom we have selected the test and control groups (Table 1).

### 2.3. Test and Control Groups

Since our application was available for free to the general public, there was a large volume of downloads by people of widely ranging commitment. To assess the effect of MITA intervention we had to identify participants who had not just downloaded MITA and used trial-and-error to arrive at solutions, but actively engaged with MITA. Thus, we have selected participants who completed at least one thousand exercises and made no more than one error per exercise (test group, N = 887). The control group was selected from the rest of participants (N = 5567) by a matching procedure. Each test group participant was matched to the control group participant by age, gender, expressive language, receptive language, sociability, cognitive awareness, and health score at first evaluation (baseline) using a propensity score analysis [26]. Therefore a total of 887 test participants and 887 control participants, selected as a result of the matching algorithm, were subjected to statistical analysis.

Average MITA use was 2.7 days/week (SE = 0.07) in the test group and 2.4 days/week (SE = 0.07) in the control group at the start of the study, Figure S1. By the end of the study MITA use decreased to 1.6 days/week (SE = 0.2) in the test group and 1.1 days/week (SE = 0.3) in the control group. These MITA use numbers represent the lower limit of the actual MITA use. The actual MITA use is likely underestimated for three reasons: (1) MITA is fully functional offline. Some devices may disconnect from the internet at some point. If they never reconnect, the MITA use data are never uploaded. (2) Whenever the app is deleted and reinstalled (so called “clean reinstall”), the un-uploaded MITA use data are completely lost. (3) When the device’s date and time have never been configured, the timestamp on puzzle records is erroneous. Puzzle records with an erroneous timestamp have been rejected. Since we can only analyze uploaded data to estimate MITA use and since we had to reject erroneous timestamp records, we have likely underestimated the actual MITA use. On the days when MITA was used, the test group used MITA on average 17.2 ± 11.3 min per day and the control group used MITA on average 13.7 ± 8.2 min per day, not counting the breaks in between exercises and not counting playtime at the end of exercises.

### 2.4. Outcome Measures

A caregiver-completed Autism Treatment Evaluation Checklist (ATEC) [22] and Mental Synthesis Evaluation Checklist (MSEC) [24] were used to track the efficacy of the treatment. The complete ATEC questionnaire is comprised of four subscales: (1) Speech/Language/Communication, (2) Sociability, (3) Sensory/Cognitive Awareness, and (4) Physical/Health/Behavior. The first subscale, Speech/Language/Communication, contains 14 items and its score ranges from 0 to 28 points. The Sociability subscale contains 20 items within a score range from 0 to 40 points. The third subscale, referred to here as the Cognitive Awareness subscale, has 18 items and scores range from 0 to 36 points. The fourth subscale, referred to here as the Health subscale, contains 25 items and scores range from 0 to 75 points. The scores from each subscale are combined in order to calculate a Total Score, which ranges from 0 to 179 points. A lower score indicates a lower severity of ASD symptoms and a higher score indicates more severe symptoms of ASD. ATEC is not a diagnostic checklist. It was designed to evaluate treatment effectiveness [22] and ASD severity can be related to ATEC total score and age only approximately. Table 2 lists the approximate ATEC total score as related to ASD severity and age as described in Mahapatra et al. [21].

ATEC was selected because it is one of the few measures validated to evaluate treatment effectiveness. In contrast, another popular ASD assessment tool, ADOS, [27] has only been validated as a diagnostic tool. Various studies confirmed the validity and reliability of ATEC [28,29,30] and several trials confirmed ATEC’s ability to longitudinally measure changes in participant performance [21,31,32,33]. Whitehouse et al. used ATEC as a primary outcome measure for a randomized controlled trial of their iPad-based intervention for ASD named TOBY, and noted ATEC’s “internal consistency and adequate predictive validity” [34]. These studies support the effectiveness of ATEC as a tool for the longitudinal tracking of symptoms and assessing changes in ASD severity.

### 2.5. Expressive Language Assessment

The ATEC Speech/Language/Communication subscale includes the following 14 items: (1) Knows own name; (2) Responds to ‘No’ or ‘Stop’; (3) Can follow some commands; (4) Can use 1 word at a time (No!, Eat, Water, etc.); (5) Can use 2 words at a time (Don’t want, Go home); (6) Can use 3 words at a time (Want more milk) and progress to interrogate complex language abilities, such as (7) Knows 10 or more words; (8) Can use sentences with 4 or more words; (9) Explains what he/she wants; (10) Asks meaningful questions; (11) Speech tends to be meaningful/relevant; (12) Often uses several successive sentences; (13) Carries on fairly good conversation; and (14) Has normal ability to communicate for his/her age. With the exception of the first three items, all the Language subscale items primarily depend on expressive language. Accordingly, the ATEC subscale 1 is referred to in this manuscript as the Expressive Language subscale to distinguish it from the Receptive Language subscale tested by the MSEC evaluation.

### 2.6. Receptive Language Assessment

MSEC evaluation was designed to be complementary to ATEC in measuring receptive language and PFS. Out of 20 MSEC items, those that directly assess receptive language are the following: (1) Understands simple stories that are read aloud; (2) Understands elaborate fairy tales that are read aloud (i.e., stories describing FANTASY creatures); (6) Understands some simple modifiers (i.e., green apple vs. red apple or big apple vs. small apple); (7) Understands several modifiers in a sentence (i.e., small green apple); (8) Understands size (can select the largest/smallest object out of a collection of objects); (9) Understands possessive pronouns (i.e., your apple vs. her apple); (10) Understands spatial prepositions (i.e., put the apple ON TOP of the box vs. INSIDE the box vs. BEHIND the box); (11) Understands verb tenses (i.e., I will eat an apple vs. I ate an apple); (12) Understands the change in meaning when the order of words is changed (i.e., understands the difference between ‘a cat ate a mouse’ vs. ‘a mouse ate a cat’); (20) Understands explanations about people, objects or situations beyond the immediate surroundings (e.g., “Mom is walking the dog,” “The snow has turned to water”). MSEC consists of 20 questions and is scored similarly to ATEC: a lower score indicates better receptive language. To simplify the interpretation of figure labels, the subscale 1 of the ATEC evaluation is referred to as the Expressive Language subscale and the MSEC scale is referred as the Receptive Language subscale.

### 2.7. Statistical Analysis

The framework for the evaluation of how the ATEC score changes over time was explained in detail in Mahapatra et al. [21]. In short, the concept of a “Visit” was developed by dividing the three-year-long observation interval into 3-month periods. All evaluations were mapped into 3-month-long bins with the first evaluation placed in the first bin. When more than one evaluation was completed within a bin, their results were averaged to calculate a single number representing this 3-month interval. It was then hypothesized that there was a two-way interaction between Visit and treatment. Statistically, this hypothesis was modeled by applying the Linear Mixed Effect Model with Repeated Measures (MMRM), where a two-way interaction term was introduced to test the hypothesis. The model (Endpoint ~ Baseline + Gender + Severity + MITA_use_nDaysPerWeek + Treatment * Visit) was fitted using the R Bioconductor library of statistical packages, in particular the “nlme” package. The subscale score at baseline, gender, severity, and MITA use measured as days per week were used as covariates. Conceptually, the model fits a plane into n-dimensional space. This plane takes into account a complex variability structure across multiple visits, including baseline differences. Once such plane is fit, and the model calculates Least Squares Means (LS Means) for each subscale and treatment group at each visit. The model also calculates LS Mean differences between the treatment and control groups at each visit. Participants in the test group were matched to those in the control group using propensity score analysis [26] based on age, gender, expressive language, receptive language, sociability, cognitive awareness, and health at the first evaluation (baseline).

### 2.8. Informed Consent

Caregivers have consented to anonymized data analysis and publication of the results.

### 2.9. Clinical Trial Registration

The observational clinical trial, ClinicalTrials.gov Identifier: NCT02708290, was registered on 15 March 2016.

### 2.10. Compliance with Ethical Standards

Using the Department of Health and Human Services regulations found at 45 CFR 46.101(b)(1), it was determined that this research project is exempt from IRB oversight.

### 2.11. Data Availability

De-identified raw data from this manuscript are available from the corresponding author upon reasonable request.

### 2.12. Code Availability Statement

Code is available from the corresponding author upon reasonable request.

## 3. Results

We first sought to replicate our earlier results [21] using the new database. The analysis of groups within the MITA database was consistent with our previous analysis performed on the database collected by the Autism Research Institute. There was no difference between females vs. males in any subscale. Younger children improved more than the older children in the Language subscale (Tables S1 and S2). Children with milder ASD improved more than children with more severe ASD in the Language subscale (Tables S3 and S4).

**Figure 4:**
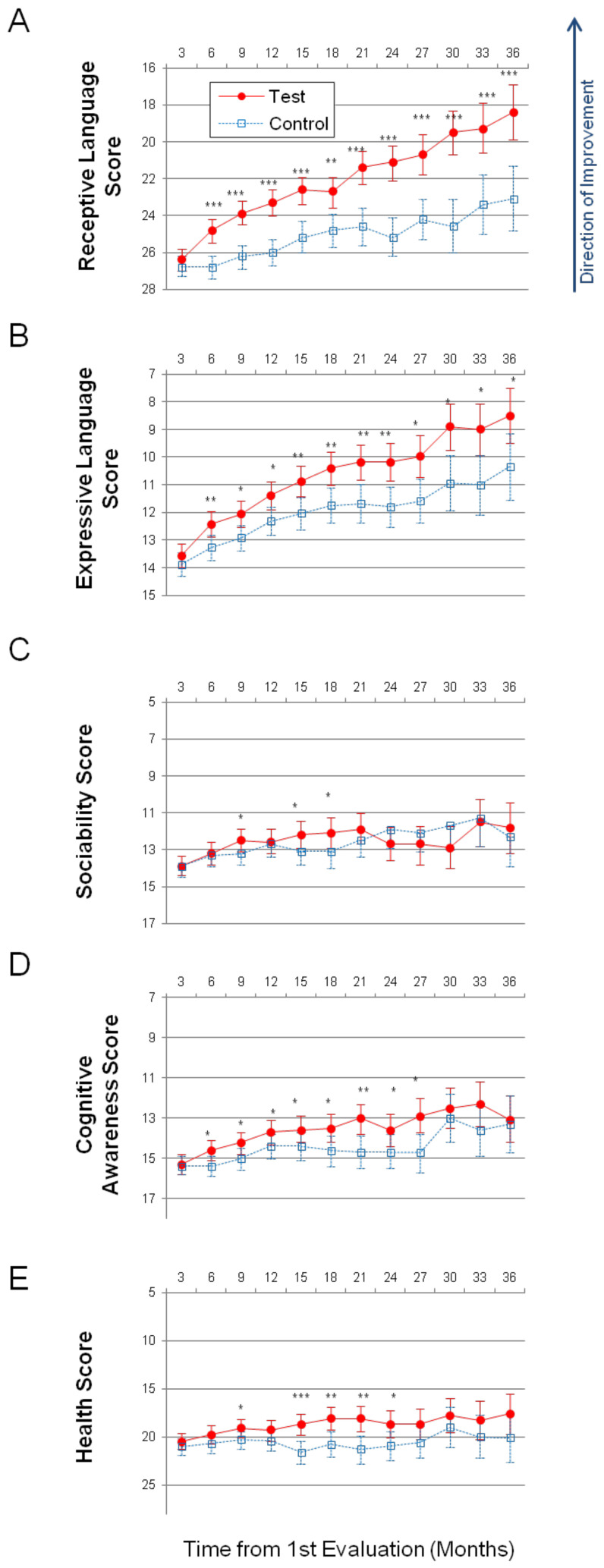
Longitudinal plots of subscale scores’ LS Means. Horizontal axis shows months from the 1st evaluation (0 to 36 months). Error bars show the 95% confidence interval. To facilitate comparison between subscales, all vertical axes ranges have been normalized to show 35% of their corresponding subscale’s maximum available score. A lower score indicates symptoms improvement. *p*-value is marked: *** < 0.0001; ** < 0.001; * < 0.05. (**A**) Receptive Language score. (**B**) Expressive Language score. (**C**) Sociability score. (**D**) Cognitive awareness score. (**E**) Health score.

Having demonstrated continuity with respect to group differences within the MITA database, we have applied the same statistical framework to study the difference between the test group and the control group (Table S5). Children in both the control and test groups improved their symptoms over time in all subscales. The greatest interest was the Receptive Language subscale targeted by the MITA intervention. The average improvement in the test group over three years was 8.01 points (SE = 0.74, *p* < 0.0001) compared to 3.70 points (SE = 0.88, *p* < 0.0001) in the control group; Figure 4A, Table 3, Table S6. The difference in the test group relative to the control group at Month 36 was statistically significant: −4.74 points (SE = 1.13, *p* < 0.0001). The negative Test – Control indicates that the test group had a lower score at month 36 and therefore milder symptoms. On the annualized basis, the test group improved their receptive language 2.2-times faster than the control group (test = 2.7 points/year; control = 1.2 points/year).

On the Expressive Language subscale, the test group improved over the three-year period by 5.07 points (SE = 0.50, *p* < 0.0001) compared to 3.55 points (SE = 0.60, <0.0001) in the control group, Figure 4B, Table S7. The difference in the test group relative to the control group at Month 36 was statistically significant: −1.84 points (SE = 0.77, *p* = 0.017). On the annualized basis, the test group improved their expressive language 1.4-times faster than the control group (test = 1.7 points/year; control = 1.2 points/year).

On the Sociability subscale, the test group improved over the three-year period by 2.06 points (SE = 0.68, *p* = 0.0026) compared to 1.65 points (SE = 0.82, *p* = 0.0444) in the control group, Figure 4C, Table S8. The difference in the test group relative to the control group at Month 36 was not statistically significant: −0.46 points (SE = 1.05, *p* = 0.6584).

On the Cognitive awareness subscale, the test group improved over the three-year period by 2.28 points (SE = 0.58, *p* < 0.0001) compared to 2.15 points (SE = 0.70, *p* = 0.0022) in the control group, Figure 4D, Table S9. The difference in the test group relative to the control group at Month 36 was not statistically significant: −0.24 points (SE = 0.9, *p* = 0.7886).

On the Health subscale, the test group improved over the three-year period by 2.89 points (SE = 1.05, *p* = 0.0059) compared to 0.83 points in the control group (SE = 1.26, *p* = 0.5102), Figure 4E, Table S10. The difference in the test group relative to the control group at Month 36 was not statistically significant: −2.57 points (SE = 1.62, *p* = 0.1119).

## 4. Discussion

In this report, we described data from a three-year-long clinical trial of PFS-targeting intervention—*Mental Imagery Therapy for Autism* or MITA [16]—that included 6454 children with ASD. This is the longest-running and the largest study of a caregiver-administered early intervention tool for young children with ASD. Both the test and control groups improved their symptoms over time in all subscales. The control participants demonstrated similar improvement to test participants in all subscales except on the Receptive Language and the Expressive Language subscales. Test participants showed a 2.2-fold greater improvement in the Receptive Language score at the end of the trial compared to the control group, Figure 4A.

### 4.1. Language Improvement Mechanisms

There are four possible explanations for the greater improvement of receptive language in MITA-engaged children, as follows: (1) evaluation bias—parents who invest more effort into the MITA app exaggerate their child’s improvement; (2) selection bias of more capable children; (3) indirect effect of MITA exercises through educating parents in the techniques of language therapy, and (4) direct effect of MITA exercises on neural networks essential for language. We discuss all four possibilities in detail below.

**1. Evaluation bias**. One possibility is that parents who have invested more time and energy into facilitating the app were also more likely to rate their child as improving. To reduce this possibility, evaluations were administered three months apart, as it is hard to remember answers to 107 questions (each question coming with three to six options) for three months. Additionally, parents were blinded to their answers in all previous evaluations. In order to examine the possibility of remaining evaluation bias, we have calculated the correlation coefficient between MITA use measured in days/week with child’s improvement in receptive language (r = −0.01), expressive language (r = −0.06), sociability (r = −0.04), cognitive awareness (r = −0.01), and health (r = 0.01). Low absolute values of correlation coefficients, as well as the variability of the direction of correlation (positive correlation for improvement of health and negative correlation for other subscales), are not consistent with the hypothesis that parents who invested more time into working with the app were also more likely to rate their child as improving. Thus, it appears that parents’ evaluation answers were not influenced by their previous evaluation answers.

**2. Selection bias.** Another possibility is that the test group participants had better capabilities in the first place, even before starting app use. To mitigate this possibility the control group participants were matched to the test group participants by age, gender, expressive language, receptive language, sociability, cognitive awareness, and health at first evaluation (baseline) using propensity score analysis. The lack of statistically significant difference at the baseline in any subscale is an indication of the successful matching procedure. Furthermore, if the children with better initial capabilities were selected into the test group, one would expect to see improved outcomes in all areas of child development. On the contrary, the test group showed a better outcome only in the Receptive Language subscale that was trained by MITA (test–control = −4.74, *p* < 0.0001) and in the related Expressive Language subscale (test–control = −1.84, *p* = 0.0172), Table 3 (the negative difference between test and control indicates that the test group had a lower score at month 36 and therefore milder symptoms). The test group outcomes in all other subscales were not statistically different from the control group; Table 3—Sociability (test–control = −0.46, *p* = 0.6584), Cognitive awareness (test–control = −0.24, *p* = 0.7886), and Health subscales (test–control = −2.57, *p* = 0.1119). Thus, selection-bias of the test group for children with better initial capabilities was not likely to be responsible for improvements in the Receptive Language score alone.

**3. Indirect effect of MITA exercises through educating parents in the techniques of language therapy.** Some caregivers could learn the language therapy techniques used in MITA directly from MITA activities or from the “Tip of the Day.” For example, parents could learn to administer directions with increasing complexity, such as: *Give me the small woodchip* -> *Give me the small white woodchip* -> *Give me two small white woodchips -> Put the small white woodchip on/under the table*, etc., and then extend these techniques to everyday activities, multiplying the effect of MITA exercises many-fold. A search of the MITA listing at the app store yielded several unsolicited MITA parents’ reviews—“MITA helps me to grab ideas from the screen and into everyday”—that supported the parents learning hypothesis. To study the parents learning hypothesis further, we have solicited feedback from MITA caregivers. More than half of responders reported that they have learned language therapy techniques from MITA exercises (unpublished observations). Moreover, the literature search revealed that even short-term parents’ interventions administered early have been shown to have significant effects [35,36] on children language acquisition, supporting the parents-learning-from-MITA-exercises hypothesis. Parsons et al. investigated the effect of a 3-month tablet-based intervention (TOBY) in an RCT of 59 children with ASD age 2 to 6 years. Improvements were shown in receptive and pragmatic language and social skills; these gains were maintained, thus suggesting skill acquisition [35]. Landa et al. investigated a 6-month intervention in 44 toddlers with ASD at age 2 years. Robust gains were observed for both IQ and communication [36]. Additionally, Huber et al. demonstrated that a short 2-month intervention substantially improved reading skills in 24 children age 7 to 12 years. This study showed rapid and widespread white matter plasticity during the reading intervention [37]. Thus, it is possible that MITA significantly affected the children’s developmental trajectory in the initial several months of the intervention, thus explaining the robust difference between the test and control groups in both Receptive and Expressive Language as early as month 6 of the study (Figure 1).

**4. Direct effect of MITA exercises.** It is possible that MITA exercises directly trained neural networks essential for language. The association of Wernicke’s and Broca’s areas with language is well-known. Less common is the realization that an understanding of full language depends on the lateral prefrontal cortex (LPFC). Wernicke’s area primarily links words with objects [38], and Broca’s area interprets the grammar and assigns words in a sentence to a grammatical group such as noun, verb, or preposition [38], but only the LPFC can synthesize the objects from memory into a novel mental image according to grammatically imposed rules [9,39]. This latter function may be called *imagination*, but we prefer a more specific term, *prefrontal synthesis* (PFS), in order to distinguish this function from other components of imagination, such as simple memory recall, dreaming, spontaneous insight, mental rotation, and integration of modifiers [1]. PFS is defined as the voluntary juxtaposition of mental objects. All MITA exercises were designed to train a child’s ability to combine mental objects. In some sense, MITA PFS exercises can be viewed as an extreme version of language therapy with minimum vocabulary training and focusing nearly exclusively on mental integration techniques. MITA exercises can also be viewed as an extension of the “matrix training” [40] with a minimal number of words and a maximum number of word combinations. We conclude that the direct effect of the MITA PFS exercises on the LPFC and its long frontoposterior connections leading to language improvement cannot be excluded, and in fact could be the most parsimonious explanation for the observed results.

### 4.2. PFS Is a Distinct Component of Executive Function

PFS was not previously identified as a separate component of executive functions. Conventionally, executive functions include inhibition and attention, working memory, and cognitive flexibility—all mediated by the LPFC [41]. PFS, defined as the process of juxtaposing multiple mental visuospatial objects, is also mediated by the LPFC [42,43,44,45,46,47]. PFS, however, is hypothesized to be mediated by a mechanism separate from other executive functions—the synchronization of object-encoding neuronal ensembles (objectNEs), whereby the LPFC phase-shifts the firing of two or more objectNEs; the synchronously firing objectNEs are perceived as a novel hybrid object (the Neuronal Ensembles Synchronization hypothesis or NES) [1,48,49]. ObjectNEs’ firing in synchrony with each other activates NMDA channels [50] and results in enhanced synapses between these objectNEs (the Hebbian “fire together–wire together”) [51]. This mechanism of PFS is different from inhibition, attention and working memory, which do not involve the synchronization of multiple objectNEs. PFS is also different from cognitive flexibility, which is commonly investigated using task-switching and set-shifting tests [41]. Such tests, e.g., Wisconsin Card Sorting Task, involve category-sorting rules identification, that do not require the synchronization of objectNEs and the visuospatial synthesis of PFS.

In addition to differences in the underlying neurology, PFS is distinguished by its phenotypic properties, such as its experience-dependent acquisition mechanism and the strong critical period. Inhibition and attention, working memory and task-switching are acquired by children independently of their use of language. Feral children and deaf linguistic isolates often exhibit normal inhibition, attention, working memory, and task-switching [8,52,53,54]. Conversely, the acquisition of PFS requires the use of recursive language in early childhood. Recursive conversations, story-telling, and fairy tales are essential for the acquisition of PFS. The myelination of frontoposterior fiber tracts mediating PFS [6] depends on early childhood conversations [7,8]. “Non-recursive” communication systems devoid of spatial prepositions and recursion, such as homesign (that spontaneously arises in families of congenitally deaf children), do not result in PFS acquisition [9].

The second important difference is the presence of the strong critical period for PFS acquisition. Inhibition and attention, working memory, and cognitive flexibility can all be improved at any age [41,55]. They have weak critical periods. PFS, however, cannot be learned after early childhood. Case studies of feral children and deaf linguistic isolates show that post-pubertal intensive therapy does not result in PFS acquisition even after many years of intervention [9]. Strong critical periods are common in the central nervous system’s development. Neural circuits underlying strong critical periods are programmed to be shaped by experience during short periods of early postnatal life; later plasticity is impossible. The PFS strong critical period has many well-studied analogs, e.g., (1) filial imprinting in birds [56], (2) monaural occlusion [57], (3) post-childbirth bonding in mammals [58], (4) the vestibulo-ocular reflex [59], (5) song dialects learning in male white-crowned sparrows [60], and (6) monocular deprivation [61]. The closure of one eye for the duration of the critical period causes a permanent loss of vision through that eye. Cats which had one eye sewn shut from birth until 3 months of age fully developed vision only in the open eye. Loss of vision occurs despite there being no damage to the sensory receptors in the eye, the thalamus, or the cerebral cortex. The simple act of covering an eye can profoundly alter the physical structure of the brain. The duration of this critical period lasts from weeks in mice to months in cats and years in primates [62].

PFS is not congruent to planning or fluid intelligence. A lot of planning is not visuospatial and therefore does not involve PFS (e.g., planning by ants, planning by bees, planning by people with PFS paralysis). Furthermore, some visuospatial scenario-playing planning occurs during REM sleep dreaming [63]. Dreaming visuospatial synthesis can be vivid and can be remembered upon waking up, thus serving its adaptive function [64]. This type of planning, however, is involuntary and occurs without the control from the LPFC (that is inactive during REM sleep [65,66]) and, therefore, is distinct from PFS, which is defined as a voluntary process completely controlled by the LPFC [67]. Finally, most PFS that occurs to understand recursive language (“the cat on the mat” vs. “the mat on the cat”) is not planning. In other words, all planning is not PFS and all PFS is not planning.

PFS is also defined more narrowly than fluid intelligence. A lot of fluid intelligence tasks can be accomplished without PFS. These tasks can be solved using amodal completion, integration of color and size, mental rotation, and patterning [39]. PFS always involves a combination of two or more mental visuospatial objects. The narrow definition of PFS is essential for uncovering its unique phenotypic characteristics, such as the experience-dependent acquisition mechanism and the strong critical period.

### 4.3. PFS Is a Distinct Component of Language

Most components of language have weak critical periods (vocabulary acquisition [68], articulation control [69], grammar processing [70], and phoneme tuning [71,72]), all of which can be significantly improved by training at any age [73,74]. PFS, however, has a strong critical period that ends between the age of five and puberty [9]. This idea was popularized by Lenneberg and is known as “Lenneberg’s language acquisition critical period hypothesis” [75]. Lenneberg’s conjecture about the strong critical period was based on a few cases of childhood traumatic aphasia and hemispherectomy. When the left hemisphere is surgically removed before the age of five (to treat cancer or epilepsy), patients often attain normal cognitive functions in adulthood (using the one remaining hemisphere). Conversely, removal of the left hemisphere after the age of five often results in PFS paralysis.

PFS ability is essential for understanding sentences describing novel combinations of objects. For example, the semantically-reversible sentences “The dog bit my friend” and “My friend bit the dog” use identical words and grammar. Appreciating the misfortune of the first sentence and the humor of the second sentence depends on the LPFC’s ability to faithfully synthesize the two objects—the friend and the dog—into a novel mental image. Similarly, the understanding of spatial prepositions such as *in, on, under, over, beside, in front of, behind* requires a subject to synthesize several objects in front of the mind’s eye. For example, the request “to put a green box {inside/behind/on top of} the blue box” requires an initial mental simulation of the scene, only after which is it possible to correctly arrange the physical objects. An inability to produce a novel mental image of the green box {inside/behind/on top of} the blue box would lead to the use of trial-and-error, which in the majority of cases will result in an incorrect arrangement.

To conduct PFS and to synchronize the objectNEs encoded in the posterior cortex (temporal, parietal, and occipital cortices), the LPFC relies on its frontoposterior connections, such as arcuate fasciculus and superior longitudinal fasciculus. Patients with damage to any component of this circuit—the LPFC [43], or the frontoposterior fibers [6], or the temporal–parietal–occipital junction [76]—often lose access to the full extent of PFS. Fuster calls their condition “prefrontal aphasia” [46] and Luria “semantic aphasia” [77]. Fuster explains that “although the pronunciation of words and sentences remains intact, language is impoverished and shows an apparent diminution of the capacity to ‘prepositionize.’ The length and complexity of sentences are reduced. There is a dearth of dependent clauses and, more generally, an underutilization of what Chomsky characterizes as the potential for recursiveness of language” (page 194). Luria reports that “these patients had no difficulty grasping the meaning of complex ideas such as ‘causation,’ ‘development,’ or ‘cooperation.’ They were also able to hold abstract conversations. However, difficulties developed when they were presented with complex grammatical constructions which coded logical relations. ... Such patients find it almost impossible to understand phrases and words which denote relative position and cannot carry out a simple instruction like ‘draw a triangle above a circle’” [77] (page 45). We prefer to call this condition ‘PFS paralysis’ since aphasia is translated from Greek as “speechless” and these patients may not experience any speech deficit.

Thus, PFS is a distinct component of both language and executive functions, different on both neurological and phenotypic levels. On the neurological level it is characterized by the LPFC-controlled phase-shift of two or more objectNEs into synchronous activity. On the phenotypic level it is characterized by a distinctly strong critical period and an unusual dependence on early childhood cultural experience: exposure to spatial prepositions, recursive conversations, story-telling, and fairytales. Furthermore, the symptoms of PFS paralysis are different from other types of aphasia, dementia, and memory deficits.

PFS paralysis is a known problem in individuals with ASD, commonly described as *stimulus overselectivity*, or *tunnel vision*, or *the lack of multi-cue responsivity* [11,12,13]. Many techniques used by speech language pathologists (SLP) and Applied Behavioral Analysis (ABA) therapists happen to aim at improving PFS. SLPs commonly refer to these techniques as “combining adjectives, location/orientation, color, and size with nouns,” “following directions with increasing complexity,” and “building the multiple features/clauses in the sentence” [78]. In ABA jargon, these techniques are known as “visual–visual and auditory–visual conditional discrimination” [79,80,81,82], “development of multi-cue responsivity” [11], and “reduction of stimulus overselectivity” [12]. MITA PFS exercises can be viewed as a special version of language therapy focusing on mental integration techniques or a type of executive functions exercises. However, the emphasis on PFS training in MITA is critical, as studying vocabulary alone, or training inhibitory function, or attention, or working memory, or set-shift alone does not result in the acquisition of PFS.

### 4.4. Why Was the MITA Effect on Language so Significant?

Test group children showed a 2.2-fold improvement in the Receptive Language score at the end of the trial vs. the control group. This result is significantly better than that reported in other trials [36,83]. There could be several reasons for the greater improvement reported herein.

(1) There may exist a synergy between MITA exercises and language therapy administered by SLPs and ABA therapists. SLP and ABA techniques also aim to improve PFS, but the PFS exercises are just a small part of language therapy that primarily focuses on building up the child’s vocabulary. Word comprehension is an easy target. It is faster to train and also highly appreciated by parents. Furthermore, most tests rely exclusively on a child’s vocabulary to measure educational success (e.g., Peabody Picture Vocabulary Test (PPVT-4) [84], Expressive Vocabulary Test (EVT-2) [85]), thus encouraging therapists to focus on vocabulary training. However, such training by itself does not train PFS that is essential for an understanding of spatial preposition, recursion, and complex language. Thus, the success of MITA intervention may derive from its exclusive focus on voluntary imagination and its most advanced component, PFS.

(2) Even when therapists administer PFS exercises, the training is mostly verbal in nature. The intuitive verbal approach is working well in neurotypical children, but can be abstruse for nonverbal and minimally verbal children with ASD. MITA, on the other hand, starts with nonverbal exercises that are much easier for children with ASD [17].

(3) Another hidden advantage of computerized language therapy for some children with ASD is the lack of personal contact in therapy and the closely related prosody stability. All MITA instructions are drawn from a pre-recorded library. Unlike human-given instructions, MITA verbal instructions are always pronounced with the same intonation. There are no variations in prosody. This prosody stability simplifies instruction interpretation for children who have auditory processing problems.

(4) The most significant challenge of conventional therapy is a substantial cost that significantly reduces therapists’ availability to most families [86]. The free MITA application, on the other hand, is always available and its everyday use does not increase the financial burden.

(5) Unlike a human therapist, MITA is readily available for download and use within minutes. As a result, parents can initiate MITA exercises when ASD diagnosis is suspected but has not been confirmed by a clinician. In fact, many MITA parents indicate their diagnosis as “suspected ASD” at registration and change their diagnosis to ASD months later, suggesting that they have started administering MITA before receiving a diagnosis from a clinician. Earlier intervention may provide an important head-start to children.

(6) To receive optimal therapy, children have to develop a certain degree of connection with a therapist [87]. In a field in which frequent rotation of therapists is a norm, a lot of time is wasted on the initial therapist–child bridge-building [88]. MITA, on the other hand, has only a minimal break-in period.

(7) Children often miss a scheduled therapy session due to travel, sickness, tantrums, or lack of focus [89]. Once a session has been missed, a therapist may not be available for a make-up lesson. One of the benefits of MITA is that parents can administer MITA anytime when they feel that children are in a good mood and receptive to therapy.

(8) When parents delegate all therapy to professionals, they may not participate in therapy sessions, and, as a result, may never learn language therapy techniques. Consequently, the most valuable time that could have been used for parent–child communication is missed. MITA inadvertently forces parents to work with their children through language exercises, therefore promoting parents’ learning of language therapy techniques.

(9) Finally, MITA gives parents hope, and it is this hope that helps parents to motivate their children and persist with language therapy. By seeing their children solving puzzles, parents become more confident of their children. As one parent wrote in an unsolicited review: “My son displays an intellectual capability, which I thought for a long time was missing.”

### 4.5. Potential Issues Associated with Use of Tablet Computers for Therapy Administration

Giving caregivers an opportunity to administer language therapy on a tablet device comes with important warnings associated with the use of a tablet. Many children left to their own devices will watch YouTube for hours. A tablet device introduces an opportunity for a caregiver to leave a child in what is perceived to be a safe and productive environment. A therapeutic app carries a significant danger of being a gateway to YouTube. Having said that, we note that tablet computers are already a common staple of most families, particularly families with minimally verbal ASD children who use tablets for communication [90]. Moreover, cognitively challenging apps such as MITA are significantly less addictive, as every step requires a mental effort. Once children run out of “cognitive energy” they lose their interest in MITA. Furthermore, MITA was designed to provide minimum sensory stimulation to reduce distractions and further lower its potential for addictiveness. It is also important to stress that every parent using MITA has signed a consent form that informed them of the danger of screen time for children and explained how to lock their tablet on the MITA application. Finally, in the future, MITA can be provided on a dedicated standalone device with no ability to download other apps so as to completely avoid the danger of exposure to YouTube and other addictive apps.

## 5. Limitations

The observational design of this study cannot definitively prove causality since unknown confounders may influence the study results. The golden standard of testing a novel clinical intervention is a randomized controlled trial (RCT). Prior to conducting the MITA study, we submitted the proposal for a therapist-administered RCT of the PFS intervention to many potential funding agencies. The proposal has failed to find any traction. We also considered a caregiver-administered RCT, but decided against it due to the high attrition rate. The only published RCT of caregiver-administered tablet-based therapy for young children with ASD reported an overwhelming drop just after 3 months, despite biweekly telephone calls to encourage app use [34]. Specifically, during the first 3-month period, participants exercised for a total median time of 1593 min (just under the recommended target of 20 min/day or 1800 min/3-month period), and during the second 3-month period, participants exercised for a total median time of 23 min (98.6% drop in app use). In effect, most participants did not receive any intervention after the first 3-month period and therefore were lost for the RCT [34]. As the minimal length of a PFS intervention RCT is likely to exceed two years [83,91], participant dropout becomes the major issue. This high attrition rate introduces multiple selection biases that devalue RCT’s ability to demonstrate causality, and essentially make it no better than an observational trial. The reported self-funded observational trial is the best study we could conduct without external funding.

Another disadvantage of the low-cost geographically diverse observational trials is their reliance on parent-reported outcome measures. There is an understanding within the psychological community that parents cannot be trusted with an evaluation of their own children. In fact, parents often yield to wishful thinking and overestimate their children’s abilities on a single assessment [92]. However, the pattern of changes can be generated by measuring the score dynamics over multiple assessments. When a single parent completes the same evaluation every three months over multiple years, changes in the score become meaningful. In this trial evaluations were administered at regular 3-month intervals.

Our results should be treated cautiously, as less motivated families may not be able to commit themselves to long-term therapy administration, and families without technical backgrounds may find their experience with MITA less intuitive. Further validation of MITA exercises is necessary to understand its efficacy within the diverse ASD population.

## 6. Conclusions

The following conclusions can be drawn from this study. (1) Prefrontal synthesis (PFS) is an essential component of full language, and exercises training PFS are an indispensable component of language therapy. (2) The MITA instructional framework is well-suited for language therapy, in terms of how stimulus is adapted automatically to a child’s performance and how performance improves over trials. (3) Intensive MITA exercises can lead to significant long-term gains in both receptive and expressive language. (4) MITA exercises can be useful for both home-based as well as schools-based language therapy delivery (many therapists currently use MITA as a reward at the end of a conventional language therapy session). (5) Caregivers are capable of administering MITA exercises to their children consistently over several years. (6) While employing MITA, caregivers learn language therapy techniques that they use elsewhere, outside of the tablet-computer environment. (7) Longitudinal parent-reported evaluations can provide objective information on children development. (8) Parent-administered and parent-reported multiyear clinical trials can be an attractive low-cost model for studying novel language, behavioral, and dietary interventions. The significant improvement in language observed in the current trial brings hope to many families and inspires us to continue developing PFS exercises and to translate MITA into multiple languages. The major strength of this study is the large number of long-term participants. The most obvious limitation is that this study’s observational design cannot definitively prove causality, since not all confounders can be adjusted appropriately. We conclude that the MITA PFS intervention warrants further investigation in a randomized controlled study.

## Figures and Tables

**Table 1:** Test and control group characteristics at the 1st evaluation (baseline). A greater ATEC score indicates greater ASD severity.

	Test	Control
Participants in each age group (total)	887	887
Age at baseline (mean ± SD)	5.3 ± 2.1	5.3 ± 2.5
Male Gender	73%	72%
ATEC Total (mean ± SD)	68.26 ± 24.14	67.73 ± 23.37

**Table 2:** Approximate relationship between ATEC total score, age, and ASD severity as described in Mahapatra et al. [21]. At any age, a greater ATEC score indicates greater ASD severity.

	Age										
Severity	2	3	4	5	6	7	8	9	10	11	12
Mild	<82	<65	<52	<43	<36	<31	<28	<25	<23	<21	<20
Moderate	82–130	65–103	52–83	43–69	36–58	31–50	28–44	25–39	23–36	21–34	20–32
Severe	130–179	103–179	83–179	69–179	58–179	50–179	44–179	39–179	36–179	34–179	32–179

**Table 3:** LS Means for test and control groups. Data are presented as LS Mean (SE; 95% CI). The differences between test and control and between month 36 and baseline are presented as LS Mean (SE; *p*-value). A lower score indicates a lower severity of ASD symptoms. The negative Test-Control relation indicates that the test group had lower score at month 36 and thus milder ASD symptoms.

	Baseline			Month 36			Month 36–Baseline	
	Test	Control	Test–Control	Test	Control	Test–Control	Test	Control
Receptive Language	26.4 (0.3; 25.8–27)	26.8 (0.29; 26.3–27.4)	−0.42 (0.29; 0.1478)	18.4 (0.75; 16.9–19.9)	23.1 (0.9; 21.4–24.9)	−4.74 (1.13; <0.0001)	−8.01 (0.74; <0.0001)	−3.7 (0.88; <0.0001)
Expressive Language	13.56 (0.22; 13.14–13.99)	13.88 (0.21; 13.47–14.3)	−0.32 (0.21; 0.12)	8.5 (0.52; 7.49–9.51)	10.34 (0.61; 9.13–11.54)	−1.84 (0.77; 0.0172)	−5.07 (0.5; <0.0001)	−3.55 (0.6; <0.0001)
Sociability	13.9 (0.28; 13.34–14.4)	13.9 (0.27; 13.41–14.5)	−0.06 (0.27; 0.83)	11.8 (0.7; 10.46–13.2)	12.3 (0.83; 10.66–13.9)	−0.46 (1.05; 0.6584)	−2.06 (0.68; 0.0026)	−1.65 (0.82; 0.0444)
Cognitive Awareness	15.3 (0.25; 14.8–15.8)	15.4 (0.25; 15–15.9)	−0.11 (0.23; 0.6506)	13.1 (0.6; 11.9–14.2)	13.3 (0.72; 11.9–14.7)	−0.24 (0.9; 0.7886)	−2.28 (0.58; <0.0001)	−2.15 (0.7; 0.0022)
Health	20.5 (0.43; 19.6–21.3)	21 (0.43; 20.1–21.8)	−0.51 (0.42; 0.2191)	17.6 (1.08; 15.5–19.7)	20.1 (1.29; 17.6–22.7)	−2.57 (1.62; 0.1119)	−2.89 (1.05; 0.0059)	−0.83 (1.26; 0.5102)

## Supplementary Materials

The following are available online at https://www.mdpi.com/2227-9032/8/4/566/s1, Table S1: MITA participants database: age groups; Table S2: MITA participants database: LS Mean differences between Age Groups; Table S3: MITA participants database, ASD severity groups; Table S4: MITA participants database: LS Mean differences between severity groups; Table S5: F-statistic and resulting p-value for the interaction term in Linear Mixed Effect Models with Repeated Measures (LMMRM) for each subscale; Table S6: LS Means (SE; 95% CI) for Receptive Language MSEC subscale score; Table S7: LS Means (SE; 95% CI) for Expressive Language measured by the Subscale 1 of ATEC; Table S8: LS Means (SE; 95% CI) for Sociability subscale score; Table S9: LS Means (SE; 95% CI) for Sensory/Cognitive Awareness subscale; Table S10: LS Means (SE; 95% CI) for Health/Physical/Behavior subscale; Figure S1: Longitudinal plot of MITA-use LS Means measured by the number of days per week when MITA exercises were administered.
