# Supplementary material for: Novel Prefrontal Synthesis Intervention Improves Language in Children with Autism
Source: Healthcare (Basel). 2020 Dec 16;8(4):566. doi: 10.3390/healthcare8040566 (PMC7765988; doi:10.3390/healthcare8040566)

Supplementary Material

Novel prefrontal synthesis intervention improves language in children with autism

Andrey Vyshedskiy[1,2*], Edward Khokhlovich, Rita Dunn[2], Alexander Faisman[2], Jonah Elgart[2], Lisa Lokshina[2], Yuriy Gankin[4], Simone Ostrovsky[2], Lauren deTorres[2], Stephen M Edelson[3], Petr Ilyinskii

ImagiRation LLC, Boston, MA, USA
Boston University, Boston, USA
Autism Research Institute, San Diego, CA, USA

Tables S1 to S4 replicate our previously published analysis (Mahapatra, Shreyas, et al. "Longitudinal Epidemiological Study of Autism Subgroups Using Autism Treatment Evaluation Checklist (ATEC) Score." *Journal of autism and developmental disorders* (2018): 1-12) on the new database of MITA participants. Since the previous analysis (performed on the database collected by the Autism Research Institute) was conducted over the two-year-period, Tables S1 to S4 only report LS Mean differences at the baseline and at the end of the two-year period (Month 24). The rest of the tables report the analysis of the three-year period from baseline to Month 36.

Table S1. MITA participants database, age groups: LS Mean differences between Month 24 and Baseline. Data are presented as LS Mean (SE; P-value). The grouping of age bands matches our previous work and is associated with uneven participants distribution over age with younger participant significantly exceeding older participants.

	**Month 24 – Baseline**		
	2-3 YOA	3-6 YOA	6-12 YOA
**Subscale 1: Language**	-7.59 (0.35; <0.0001)	-4.23 (0.21; <0.0001)	-1.24 (0.39; 0.47)
**Subscale 2: Sociability**	-2.23 (0.48; <0.004)	-2.30 (0.29; <0.0001)	-1.43 (0.54; 0.88)
**Subscale 3: Cognitive Awareness**	-2.8 (0.41; <0.0001)	-1.60 (0.24; <0.0001)	-0.17 (0.47; 1.00)
**Subscale 4: Health**	0.08 (0.76; 1.00)	-1.75 (0.46; 0.08)	-0.62 (0.86; 1.00)

Table S2. MITA participants database, LS Mean differences between Age Groups: Data are presented as: LS Mean difference (SE; P-Value)

	Baseline			Month 24		
	2-3 vs. 3-6	2-3 vs. 6-12	3-6 vs. 6-12	2-3 vs. 3-6	2-3 vs. 6-12	3-6 vs. 6-12
**Subscale 1: Language**	1.14 (0.20; <0.0001)	1.61 (0.28; <0.0001)	0.47 (0.22; 0.99)	-2.22 (0.39; <0.0001)	-4.73 (0.52; <0.0001)	-2.51 (0.43; <0.0001)
**Subscale 2: Sociability**	0.75 (0.26; <0.72)	1.10 (0.35; <0.55)	0.35 (0.28; 1.00)	0.82 (0.53; 1.00)	0.29 (0.70; 1.00)	-0.52 (0.58; 1.00)
**Subscale 3: Cognitive Awareness**	0.75 (0.22; 0.32)	0.86 (0.31; 0.76)	0.11 (0.24; 1.00)	-0.46 (0.46; 1.00)	-1.77 (0.60; 0.66)	-1.32 (0.50; 0.89)
**Subscale 4: Health**	0.40 (0.42; 1.00)	0.78 (0.58; 1.00)	0.38 (0.46; 1.00)	2.23 (0.85; 0.88)	1.48 (1.12; 1.00)	--0.75 (0.94; 1.00)

Table S3. MITA participants database, ASD severity groups: LS Mean differences between Month 24 and Baseline. Data are presented as LS Mean (SE; P-value).

	Month 24 – Baseline		
	Mild	Moderate	Severe
**Subscale 1: Language**	-6.23 (0.35; <0.0001)	-4.90 (0.26; <0.0001)	-2.91 (0.26; <0.0001)
**Subscale 2: Sociability**	-0.47 (0.48; 1.00)	-2.03 (0.36; <0.0001)	-3.16 (0.35; <0.0001)
**Subscale 3: Cognitive Awareness**	interaction term not significant	interaction term not significant	interaction term not significant
**Subscale 4: Health**	1.43 (0.76; <0.99)	-0.27 (0.57; 1.00)	-3.53 (0.56; <0.0001)

Table S4. MITA participants database: LS Mean differences between severity groups. Data are presented as: LS Mean difference (SE; P-Value)

	Baseline			Month 24		
	Mild vs. Moderate	Mild vs. Severe	Moderate vs. Severe	Mild vs. Moderate	Mild vs. Severe	Moderate vs. Severe
**Subscale 1: Language**	-0.52 (0.21; 0.87)	-0.41 (0.23; 0.99)	0.11 (0.18; 1.00)	-1.85 (0.42; 0.006)	-3.73 (0.43; <0.0001)	-1.88 (0.36; 0.0001)
**Subscale 2: Sociability**	-2.53 (0.28; <0.0001)	-3.95 (0.32; <0.0001	-1.42 (0.24; <0.0001)	0.97 (0.57; 0.99)	-1.26 (0.59; 0.98)	-0.28 (0.47; 1.00)
**Subscale 3: Cognitive Awareness**	interaction term not significant	interaction term not significant	interaction term not significant	interaction term not significant	interaction term not significant	interaction term not significant
**Subscale 4: Health**	-3.16 (0.44; <0.0001)	-5.97 (0.52; <0.0001)	-2.81 (0.39; <0.0001)	-1.46 (0.90; 1.0000)	-1.01 (0.94; 1.00)	-0.45 (0.78; 1.00)

Table S5. F-statistic and resulting p-value for the interaction term in Linear Mixed Effect Models with Repeated Measures (LMMRM) for each subscale

	F-value	p-value
**Receptive Language**	7.580	<.0001
**Expressive Language**	2.357	0.0067
**Sociability**	1.532	0.1127
**Cognitive Awareness**	1.76	0.0556
**Health**	1.880	0.0371

Table S6. LS Means (SE; 95% CI) for Receptive Language MSEC subscale score. The differences between test and control and between Month 36 and Baseline are presented as LS Mean (SE; P-value). A lower score indicates lower severity of ASD symptoms. The negative Test-Control indicates that the Test group had lower score and therefore milder symptoms.

Visit Number	Test	Control	Test - Control
Baseline	26.4 (0.3; 25.8 - 27)	26.8 (0.29; 26.3 - 27.4)	-0.42 (0.29; 0.1478)
Month 6	24.8 (0.33; 24.2 - 25.5)	26.8 (0.33; 26.2 - 27.4)	-1.97 (0.36; <0.0001)
Month 9	23.9 (0.34; 23.2 - 24.5)	26.2 (0.34; 25.5 - 26.8)	-2.3 (0.37; <0.0001)
Month 12	23.3 (0.36; 22.6 - 24)	26.0 (0.37; 25.3 - 26.7)	-2.7 (0.42; <0.0001)
Month 15	22.6 (0.39; 21.9 - 23.4)	25.2 (0.42; 24.4 - 26.1)	-2.6 (0.49; <0.0001)
Month 18	22.7 (0.43; 21.9 - 23.6)	24.8 (0.47; 23.9 - 25.7)	-2.08 (0.56; 0.0002)
Month 21	21.4 (0.47; 20.5 - 22.3)	24.6 (0.51; 23.6 - 25.6)	-3.2 (0.63; <0.0001)
Month 24	21.1 (0.49; 20.2 - 22.1)	25.2 (0.54; 24.2 - 26.3)	-4.08 (0.66; <0.0001)
Month 27	20.7 (0.56; 19.6 - 21.8)	24.2 (0.58; 23.1 - 25.3)	-3.54 (0.75; <0.0001)
Month 30	19.5 (0.62; 18.3 - 20.7)	24.6 (0.74; 23.2 - 26.1)	-5.13 (0.92; <0.0001)
Month 33	19.3 (0.7; 17.9 - 20.6)	23.4 (0.8; 21.8 - 25)	-4.13 (1.02; <0.0001)
Month 36	18.4 (0.75; 16.9 - 19.9)	23.1 (0.9; 21.4 - 24.9)	-4.74 (1.13; <0.0001)
Month 36 - Baseline	-8.01 (0.74; <0.0001)	-3.7 (0.88; <0.0001)	na

Table S7. LS Means (SE; 95% CI) for Expressive Language measured by the Subscale 1 of ATEC. The differences between Test and Control and between Month 36 and Baseline are presented as LS Mean (SE; P-value). A lower score indicates lower severity of ASD symptoms.

Visit Number	Test	Control	Test - Control
Baseline	13.56 (0.22; 13.14 - 13.99)	13.88 (0.21; 13.47 - 14.3)	-0.32 (0.21; 0.12)
Month 6	12.42 (0.24; 11.96 - 12.88)	13.27 (0.23; 12.81 - 13.73)	-0.85 (0.25; 0.0006)
Month 9	12.06 (0.24; 11.59 - 12.53)	12.92 (0.24; 12.45 - 13.39)	-0.86 (0.26; 0.001)
Month 12	11.39 (0.26; 10.88 - 11.89)	12.31 (0.26; 11.8 - 12.82)	-0.92 (0.29; 0.0016)
Month 15	10.88 (0.28; 10.33 - 11.43)	12.03 (0.29; 11.45 - 12.6)	-1.15 (0.34; 0.0007)
Month 18	10.41 (0.31; 9.82 - 11.01)	11.75 (0.33; 11.12 - 12.39)	-1.34 (0.39; 0.0005)
Month 21	10.18 (0.33; 9.55 - 10.82)	11.68 (0.36; 10.98 - 12.38)	-1.49 (0.43; 0.0005)
Month 24	10.18 (0.34; 9.51 - 10.85)	11.8 (0.37; 11.07 - 12.52)	-1.62 (0.45; 0.0004)
Month 27	9.98 (0.39; 9.22 - 10.74)	11.59 (0.4; 10.8 - 12.38)	-1.61 (0.51; 0.0018)
Month 30	8.91 (0.43; 8.08 - 9.75)	10.94 (0.51; 9.95 - 11.94)	-2.03 (0.63; 0.0012)
Month 33	9 (0.48; 8.06 - 9.94)	11.01 (0.55; 9.94 - 12.09)	-2.01 (0.69; 0.0038)
Month 36	8.5 (0.52; 7.49 - 9.51)	10.34 (0.61; 9.13 - 11.54)	-1.84 (0.77; 0.0172)
Month 36 - Baseline	-5.07 (0.5; <0.0001)	-3.55 (0.6; <0.0001)	na

Table S8. LS Means (SE; 95% CI) for Sociability subscale score. The differences between Test and Control and between Month 36 and Baseline are presented as LS Mean (SE; P-value).

Visit Number	Test	Control	Test - Control
Baseline	13.9 (0.28; 13.34 - 14.4)	13.9 (0.27; 13.41 - 14.5)	-0.06 (0.27; 0.83)
Month 6	13.2 (0.31; 12.57 - 13.8)	13.3 (0.3; 12.68 - 13.9)	-0.1 (0.33; 0.7618)
Month 9	12.5 (0.32; 11.89 - 13.1)	13.2 (0.31; 12.62 - 13.8)	-0.72 (0.34; 0.0361)
Month 12	12.6 (0.34; 11.89 - 13.2)	12.7 (0.34; 12.03 - 13.4)	-0.15 (0.39; 0.6953)
Month 15	12.2 (0.37; 11.43 - 12.9)	13.1 (0.39; 12.31 - 13.8)	-0.93 (0.45; 0.042)
Month 18	12.1 (0.41; 11.27 - 12.9)	13.1 (0.44; 12.29 - 14)	-1.08 (0.52; 0.0378)
Month 21	11.9 (0.43; 11.04 - 12.7)	12.5 (0.48; 11.52 - 13.4)	-0.57 (0.58; 0.3252)
Month 24	12.7 (0.46; 11.79 - 13.6)	11.9 (0.5; 10.96 - 12.9)	0.75 (0.61; 0.2233)
Month 27	12.7 (0.52; 11.71 - 13.8)	12.1 (0.54; 11.01 - 13.1)	0.67 (0.7; 0.3373)
Month 30	12.9 (0.57; 11.75 - 14)	11.7 (0.69; 10.32 - 13)	1.2 (0.85; 0.1573)
Month 33	11.5 (0.65; 10.25 - 12.8)	11.3 (0.74; 9.88 - 12.8)	0.18 (0.94; 0.8528)
Month 36	11.8 (0.7; 10.46 - 13.2)	12.3 (0.83; 10.66 - 13.9)	-0.46 (1.05; 0.6584)
Month 36 - Baseline	-2.06 (0.68; 0.0026)	-1.65 (0.82; 0.0444)	na

Table S9. LS Means (SE; 95% CI) for Sensory/Cognitive Awareness subscale. The differences between Test and Control and between Month 36 and Baseline are presented as LS Mean (SE; P-value).

Visit Number	Test	Control	Test - Control
Baseline	15.3 (0.25; 14.8 - 15.8)	15.4 (0.25; 15 - 15.9)	-0.11 (0.23; 0.6506)
Month 6	14.6 (0.27; 14.1 - 15.1)	15.4 (0.27; 14.9 - 15.9)	-0.77 (0.29; 0.0068)
Month 9	14.2 (0.28; 13.7 - 14.8)	15 (0.28; 14.4 - 15.5)	-0.78 (0.3; 0.0097)
Month 12	13.7 (0.3; 13.1 - 14.3)	14.4 (0.3; 13.8 - 15)	-0.74 (0.34; 0.0282)
Month 15	13.6 (0.32; 12.9 - 14.2)	14.4 (0.34; 13.7 - 15.1)	-0.84 (0.39; 0.0324)
Month 18	13.5 (0.35; 12.8 - 14.2)	14.6 (0.38; 13.8 - 15.3)	-1.09 (0.45; 0.0152)
Month 21	13 (0.38; 12.3 - 13.8)	14.7 (0.42; 13.9 - 15.5)	-1.65 (0.5; 0.0009)
Month 24	13.6 (0.4; 12.8 - 14.4)	14.7 (0.43; 13.9 - 15.6)	-1.17 (0.53; 0.0268)
Month 27	12.9 (0.45; 12 - 13.7)	14.7 (0.47; 13.7 - 15.6)	-1.81 (0.6; 0.0026)
Month 30	12.5 (0.5; 11.5 - 13.5)	13 (0.59; 11.8 - 14.2)	-0.5 (0.73; 0.4944)
Month 33	12.3 (0.56; 11.2 - 13.4)	13.6 (0.64; 12.3 - 14.8)	-1.22 (0.81; 0.1336)
Month 36	13.1 (0.6; 11.9 - 14.2)	13.3 (0.72; 11.9 - 14.7)	-0.24 (0.9; 0.7886)
Month 36 - Baseline	-2.28 (0.58; <0.0001)	-2.15 (0.7; 0.0022)	na

Table S10. LS Means (SE; 95% CI) for Health/Physical/Behavior subscale. The differences between Test and Control and between Month 36 and Baseline are presented as LS Mean (SE; P-value).

Visit Number	Test	Control	Test - Control
Baseline	20.5 (0.43; 19.6 - 21.3)	21 (0.43; 20.1 - 21.8)	-0.51 (0.42; 0.2191)
Month 6	19.8 (0.48; 18.8 - 20.7)	20.7 (0.47; 19.7 - 21.6)	-0.9 (0.51; 0.0758)
Month 9	19.1 (0.49; 18.2 - 20.1)	20.3 (0.49; 19.3 - 21.2)	-1.14 (0.54; 0.0341)
Month 12	19.3 (0.52; 18.3 - 20.4)	20.4 (0.53; 19.4 - 21.5)	-1.08 (0.6; 0.0721)
Month 15	18.7 (0.57; 17.6 - 19.8)	21.6 (0.6; 20.4 - 22.8)	-2.88 (0.7; <0.0001)
Month 18	18.1 (0.63; 16.9 - 19.3)	20.8 (0.67; 19.5 - 22.2)	-2.73 (0.8; 0.0007)
Month 21	18.1 (0.67; 16.8 - 19.4)	21.3 (0.74; 19.8 - 22.7)	-3.14 (0.89; 0.0004)
Month 24	18.7 (0.7; 17.3 - 20.1)	20.9 (0.77; 19.4 - 22.4)	-2.18 (0.95; 0.0214)
Month 27	18.7 (0.81; 17.1 - 20.3)	20.6 (0.84; 19 - 22.3)	-1.96 (1.08; 0.0682)
Month 30	17.8 (0.89; 16 - 19.5)	19 (1.06; 16.9 - 21.1)	-1.22 (1.31; 0.3511)
Month 33	18.3 (1; 16.3 - 20.3)	20 (1.15; 17.8 - 22.3)	-1.7 (1.45; 0.2424)
Month 36	17.6 (1.08; 15.5 - 19.7)	20.1 (1.29; 17.6 - 22.7)	-2.57 (1.62; 0.1119)
Month 36 - Baseline	-2.89 (1.05; 0.0059)	-0.83 (1.26; 0.5102)	na

Figure S1. Longitudinal plot of MITA-use LS Means measured by the number of days per week when MITA exercises were administered. Horizontal axis shows months from the 1st evaluation (0 to 36 months). Error bars show the 95% confidence interval. P-value is marked: **<0.001; *<0.05.

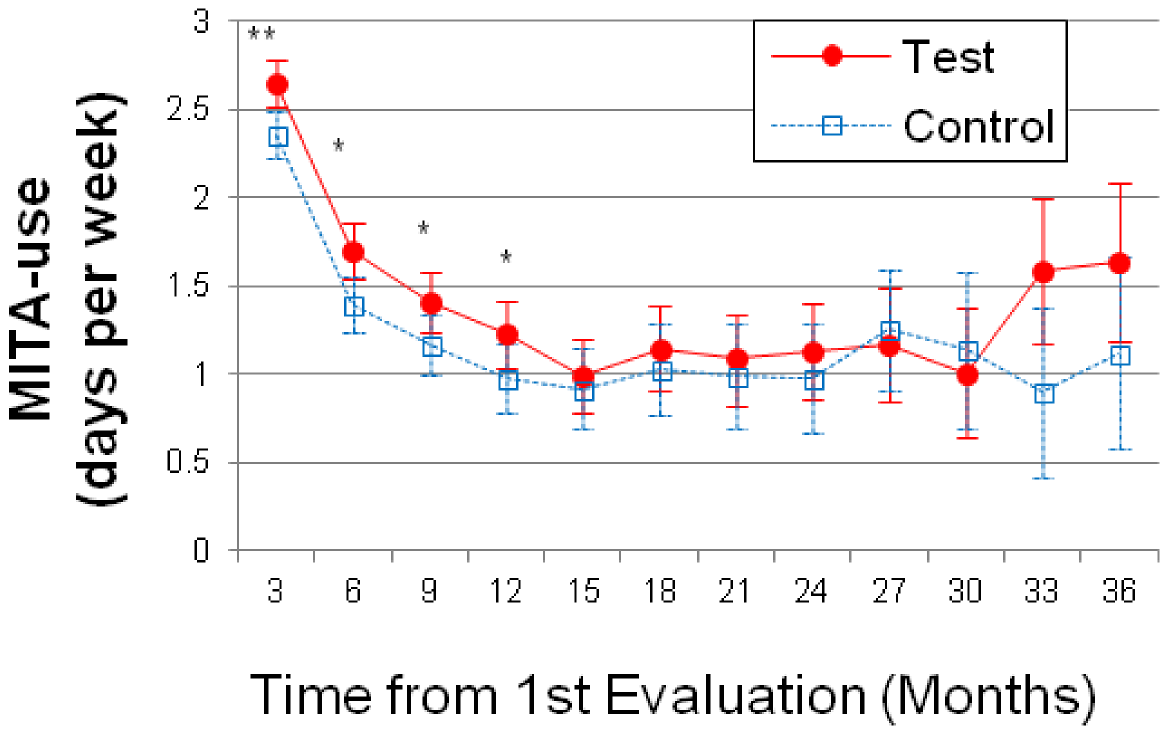

Supplement: Supplementary file 1 [file healthcare-08-00566-s001.pdf]
